# Airway mir-155 responses are associated with TH1 cytokine polarization in young children with viral respiratory infections

**DOI:** 10.1371/journal.pone.0233352

**Published:** 2020-05-22

**Authors:** Maria Arroyo, Kyle Salka, Elizabeth Chorvinsky, Xilei Xuchen, Karima Abutaleb, Geovanny F. Perez, Jered Weinstock, Susana Gaviria, Maria J. Gutierrez, Gustavo Nino

**Affiliations:** 1 Department of Pediatrics, Division of Pediatric Pulmonary and Sleep Medicine, Children’s National Medical Center, George Washington University, Washington, DC, United States of America; 2 Department of Pediatrics, Division of Pediatric Pulmonology, University at Buffalo, The State University of New York, Buffalo, NY, United States of America; 3 Division of Pediatric Allergy and Immunology, Johns Hopkins University, Baltimore, MD, United States of America; Center for Disease Control and Prevention, UNITED STATES

## Abstract

**Background:**

MicroRNAs (miRs) control gene expression and the development of the immune system and antiviral responses. MiR-155 is an evolutionarily-conserved molecule consistently induced during viral infections in different cell systems. Notably, there is still an unresolved paradox for the role of miR-155 during viral respiratory infections. Despite being essential for host antiviral TH1 immunity, miR-155 may also contribute to respiratory disease by enhancing allergic TH2 responses and NFkB-mediated inflammation. The central goal of this study was to define how airway miR-155 production is related to TH1, TH2, and pro-inflammatory cytokine responses during naturally occurring viral respiratory infections in young children.

**Methods:**

Normalized nasal airway levels of miR-155 and nasal protein levels of IFN-γ, TNF-α, IL-1β, IL-13, IL-4 were quantified in young children (≤2 years) hospitalized with viral respiratory infections and uninfected controls. These data were linked to individual characteristics and respiratory disease parameters.

**Results:**

A total of 151 subjects were included. Increased miR-155 levels were observed in nasal samples from patients with rhinovirus, RSV and all respiratory viruses analyzed. High miR-155 levels were strongly associated with high IFN-γ production, increased airway TH1 cytokine polarization (IFN-γ/IL-4 ratios) and increased pro-inflammatory responses. High airway miR-155 levels were linked to decreased respiratory disease severity in individuals with high airway TH1 antiviral responses.

**Conclusions:**

The airway secretion of miR-155 during viral respiratory infections in young children is associated with enhanced antiviral immunity (TH1 polarization). Further studies are needed to define additional physiological roles of miR-155 in the respiratory tract of human infants and young children during health and disease.

## Introduction

MicroRNAs (miRNAs) are small non-coding RNA molecules that control gene expression and critically regulate the development of the immune system and antiviral responses [1,2]. Notably, one of the most investigated miRNAs in immunology and virology is miR-155 [3–6]. The interest in this molecule was initially motivated by the discovery that miR-155 is the only miRNA substantially induced by the synthetic viral intermediate poly(I:C) or the host antiviral interferon (IFN) response in macrophages [7]. Over the past decade many studies in humans and animal models have confirmed that miR-155 is an evolutionarily-conserved molecule consistently induced during viral infections in different cell systems [7–12]. Moreover, miR-155 has also been reported to have potent antiviral actions including the activation of CD4+ and CD8+ T cell responses [8–10], the inhibition of SOCS1, the negative regulation of JAK/STAT and IFN signaling [13], and the regulation of multiple TLRs including TLR2, TLR3, TLR4, TLR7 and TLR8 [1,2]. As a result, miR-155 has been shown to enhance TH1 antiviral responses against viruses *in vivo* [8,12].

Studies in human infants conducted by our team [14] and others [15] have identified abundant airway production of miR-155 during naturally occurring infections caused by rhinovirus (RV) or respiratory syncytial virus (RSV). However, there is still an important unresolved paradox for the role of miR-155 during viral respiratory infections. Despite being essential for the generation of host antiviral TH1 immunity [3–12], miR-155 may also contribute to respiratory disease by enhancing allergic TH2 responses [16–19] and NFkB-mediated inflammation in macrophages and other bone marrow-derived immune cells [20–23]. Recent studies have shown that miR-155 knockout (KO) mice have decreased lung inflammation and recover faster from influenza infection [22]. Similarly, cigarette smoke-induced lung inflammation is reduced in miR-155 KO mice and mitigated by anti-miR-155 treatment [23]. Collectively, these data demonstrate that miR-155 has a powerful but unclear role fine-tuning host TH1/TH2 antiviral immunity. Human-based studies are still needed to better understand the protective and/or pathogenic role(s) of miR-155 during viral respiratory infections.

The central goal of this study was to define, for the first time in human infants, how airway miR-155 production is related to TH1, TH2, and pro-inflammatory cytokine responses during naturally occurring viral respiratory infections. Our results provide novel evidence that high miR-155 levels are associated with the presence of strong TH1 cytokine polarization (IFN-γ/ IL-4 ratios) as well as increased airway pro-inflammatory responses. These novel data highlight the importance of investigating the secretory responses of miR-155 in the respiratory tract of young children. The latter can have high impact since miR-155 may play a critical regulatory role for both:1) the generation of protective host antiviral immunity; and 2) the balance of TH1/TH2 inflammatory responses during viral respiratory infections that occur in early life.

## Methods

### Study population

Young children (≤2 years; n = 140) were recruited during hospitalization due to PCR-confirmed viral respiratory infection at Children’s National Health System (CNHS) in Washington, DC. We included age-matched controls without viral respiratory infection (negative viral PCR) recruited during non-respiratory hospitalizations or outpatient/emergency department visits (n = 11). Characteristics of the study subjects are presented in Table 1. All clinical and demographic variables were obtained by reviewing electronic medical records (EMR) at CNHS. The Institutional Review Board (IRB) of CNHS approved the study and granted a waiver of informed consent given that this research involved materials (data, documents, records, or specimens) collected solely for non-research purposes (clinical indications).

**Table 1:** Nasal airway miR-155 levels according to respiratory viruses in young children. RSV = human respiratory syncytial virus, RV = human rhinovirus, HMPV = human metapneumovirus. *Virus frequency includes single and mixed viral infections. ** miR-155 levels calculated with ΔΔCt method normalized to a spike in control (cel-miR-39) and presented as median and IQR. ***p values relative to miR-155 values in the control (uninfected) group. No significant differences were observed in miR-155 levels according to virus (Kruskal Wallis p = 0.83).

Virus	N*	Nasal miR-155 levels**	p-value***
**Control**	11	1.33 (0.1–3.8)	Reference
**All viruses**	140	7.04 (1.6–37.9)	0.004
**RSV**	32	10.8 (1.4–34)	0.008
**RV**	82	4.56 (1.3–32.8)	0.014
**Adenovirus**	19	14.2 (3.4–234)	0.004
**Parainfluenza**	10	3.53 (1.2–21.8)	0.14
**Influenza**	10	25.3 (3.6–94)	0.04
**HMPV**	22	5.8 (1.3–39)	0.02
**Mixed**	36	4.54 (1.7–46.5)	0.01

### Nasal wash collection, viral PCR analysis, and cytokine measurements

Nasal secretions collected at the onset of acute respiratory illnesses for diagnostic purposes were analyzed by a viral multiplex PCR panel for 12 targets (rhinovirus (RV), RSV A, RSV B, Human Metapneumovirus (HMPV), parainfluenza 1–3, influenza A and B, H1N1, H1N3, Adenovirus) (Luminex, TX, USA). Nasal protein levels of IFN-γ, TNF-α, IL-1β, IL-13, and IL-4 were quantified by electrochemiluminescence (MesoScale, MD, USA).

### Quantification of nasal airway miR-155 levels

Total RNA was first extracted from the nasal secretions using MirVana miRNA isolation kit (Invitrogen, AM1560), following enrichment procedure for small RNAs as per manufacturer’s protocol. For normalization we added a spike-in (Cel-mir-39-3p, sequence 5’-UCACCGGGUGUAAAUCAGCUUG-3’, ThermoFisher miRNA mimic, Assay ID# MC10956, cat# 4464066) to the viral nasal wash prior to mirVana mirna isolation protocol. Extracted RNA was then reverse transcribed into cDNA. Quantitative real-time RT-PCR was used to measure miR-155 and Cel-mir-39 levels (TaqMan assay, ThermoFisher, MA, USA). The following primers were used: Cel-mir-39 primer (ThermoFisher MicroRNA assay, Assay ID: 000200) Stem-loop sequence of primer 5’UAUACCGAGAGCCCAGCUGAUUUCGUCUUGGUAAUAAGCUCGUCAUUGAGAUUAUCACCGGGUGUAAAUCAGCUUGGCUCUGGUGUC-3’

HSA-mir-155 primer (ThermoFisher MicroRNA assay, Assay ID: 002623)

Stem-loop sequence of primer 5’UUAAUGCUAAUCGUGAUAGGGGUUGUUCUUAUUAACAGACACCUAACAUGUUAGCAUUAGCU-3’

The delta Ct (ΔΔCt) method was used to calculate final miR-155 levels normalized to a spike-in control (cel-mir-39, ThermoFisher, MA, USA).

### Clinical and demographic variables

We collected demographic and clinical information from EMR to evaluate disease severity including wheezing, retractions, supplemental O2 needs, respiratory rate, and heart rate at the initial presentation. We combined these parameters into a composite respiratory distress score as described [24] (S1 Table).

### Data analysis

Descriptive statistics included Chi-square test for binary variables and non-parametric tests (Wilcoxon test) for continuous variables. Comparisons of miR-155 levels and cytokines across groups were done with Kruskal-Wallis test or Wilcoxon test as appropriate. Linear regression models (univariate and multivariate) were built to examine the association between miR-155 levels and other molecular/clinical predictors with respiratory severity scores at initial presentation. Significance was taken at the P < 0.05 level. (V.19 Minitab, Inc., PA, USA).

## Results

### Airway production of miR-155 during viral respiratory infection in early life

A total of 151 subjects were included in this study. We found that relative to uninfected controls (n = 11), young children (≤2 years) hospitalized with viral respiratory infection (n = 140) had significantly increased nasal levels of miR-155 (Table 1). No significant differences were observed according to viral pathogen or mixed infections (Table 1). All viruses had increased miR-155 levels relative to controls (uninfected group), although Parainfluenza did not reach statistical significance likely due to the small number of cases (Table 1).

Baseline characteristics of all study subjects are shown in Table 2. Due to the presence of non-parametric distribution and extreme miR-155 values, we used quartiles to define groups of children with low (<25th percentile), medium (25-50th percentile) or high (>75th percentile) airway miR-155 levels. We did not identify significant differences in demographics or baseline clinical characteristics among the individuals with low, medium or high airway miR-155 levels during viral respiratory infections (Table 2).

**Table 2:** Baseline characteristics of study subjects: VRI = viral respiratory infection, RSV = human respiratory syncytial virus, RV = human rhinovirus, HMPV = human metapneumovirus. *Percentage of each virus includes single and mixed viral infections.**p values obtained by Kruskal-Wallis test for continuous variables and logistic regression for binary variables (RV reference group for viral pathogens).

Variable	Controls	VRI High miR-155	VRI Medium miR-155	VRI Low miR-155	**p-value
**Total number of subjects, n**	11	35	70	35	-
**Age in years, median**	0.81	0.99	1.13	1.11	0.36
**Male (%)**	63	60	65	60	0.94
**Black race/ethnicity (%)**	64	46	59	66	0.77
**Preterm (%<37 weeks gestation)**	40	31	50	48	0.18
Viral pathogen *					
**RV (%)**	-	63	60	51	0.59
**RSV (%)**	-	25	23	20	0.94
**HMPV (%)**	-	17	16	14	0.85
**Parainfluenza (%)**	-	9	9	3	0.52
**Influenza (%)**	-	3	7	11	0.77
**Adenovirus (%)**	-	6	13	23	0.10
**Mixed (%)**	-	23	25	28	0.97

### MiR-155 production is associated with stronger airway TH1 and pro-inflammatory responses in young children with viral respiratory infections

**Fig 1:**
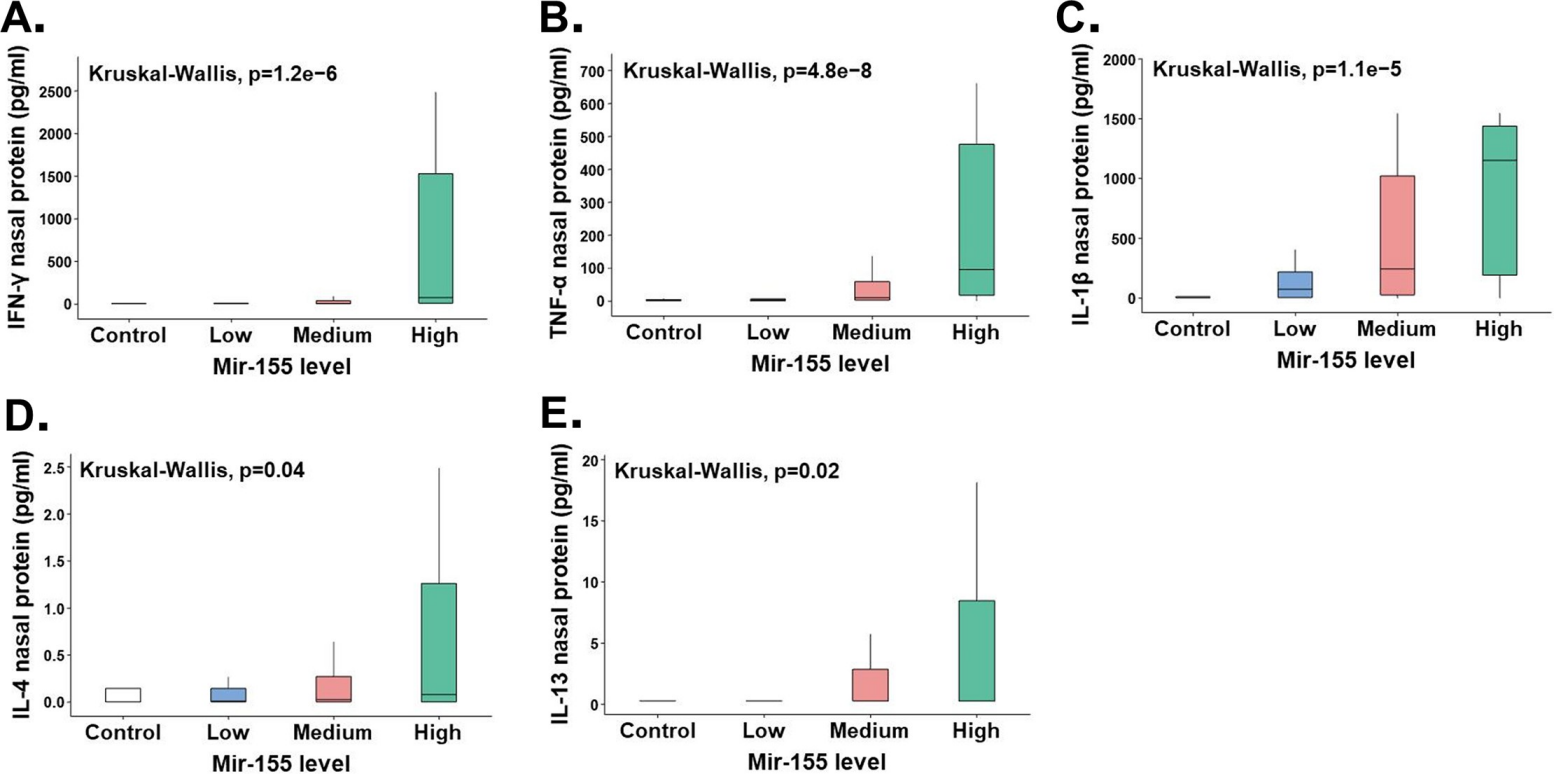
Nasal miR-155 levels stratified as high (>75th%ile), medium (25-75th%ile), or low (<25th%ile) demonstrate that young children with high miR-155 levels during viral respiratory infections (green boxplots) have higher airway production of (A) IFN-γ, (C) TNF- α, (C) IL-1β, (D) IL-4 and (E) IL-13.

**Fig 2:**
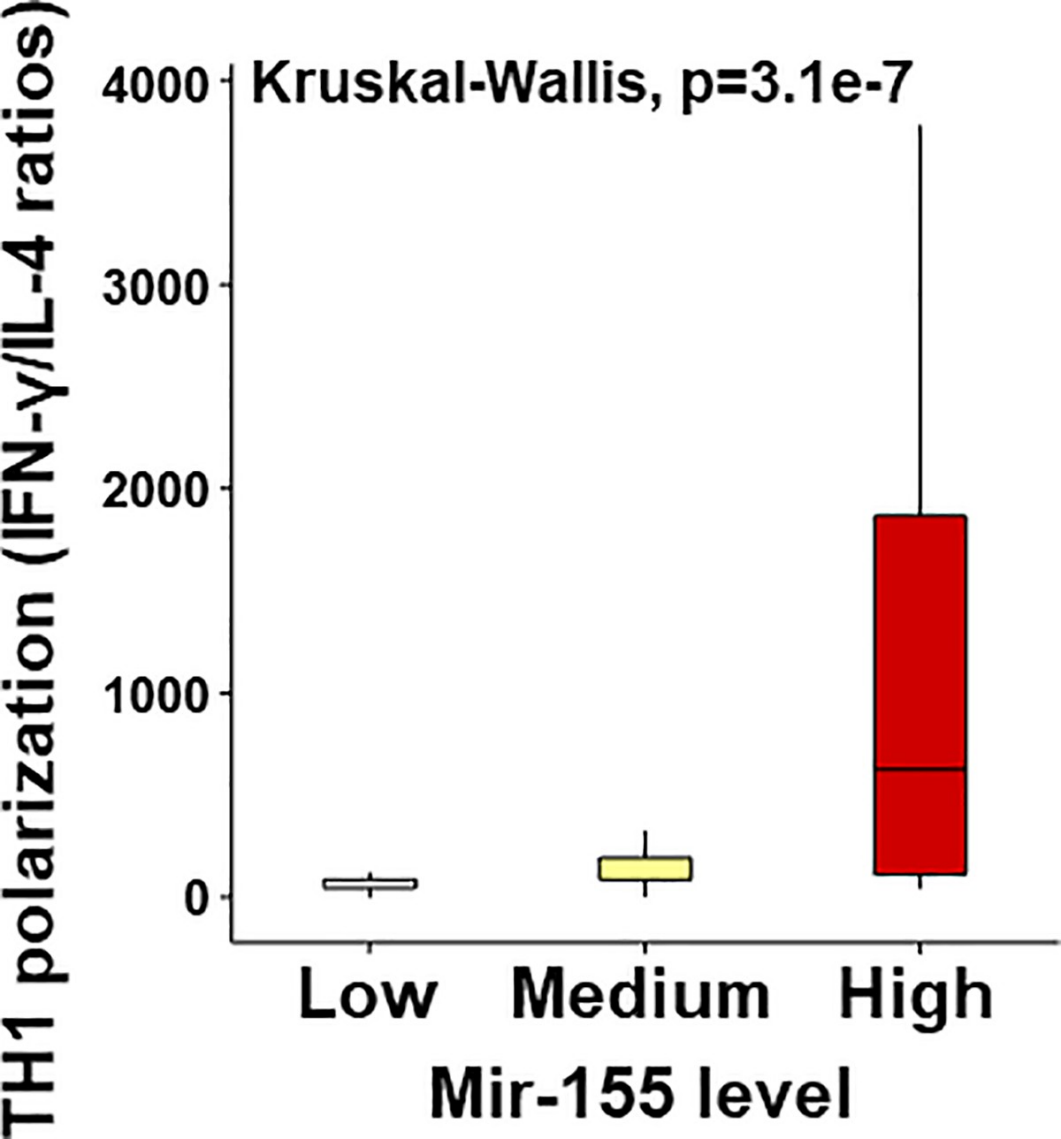
Nasal miR-155 levels stratified as high (>75th%ile), medium (25-75th%ile), or low (<25th%ile) demonstrate that young children with high miR-155 levels during viral respiratory infections (green boxplots) have higher airway TH1 cytokine polarization (IFN-γ:IL-4 ratios, calculated with protein concentration pg/ml).

**Fig 3:**
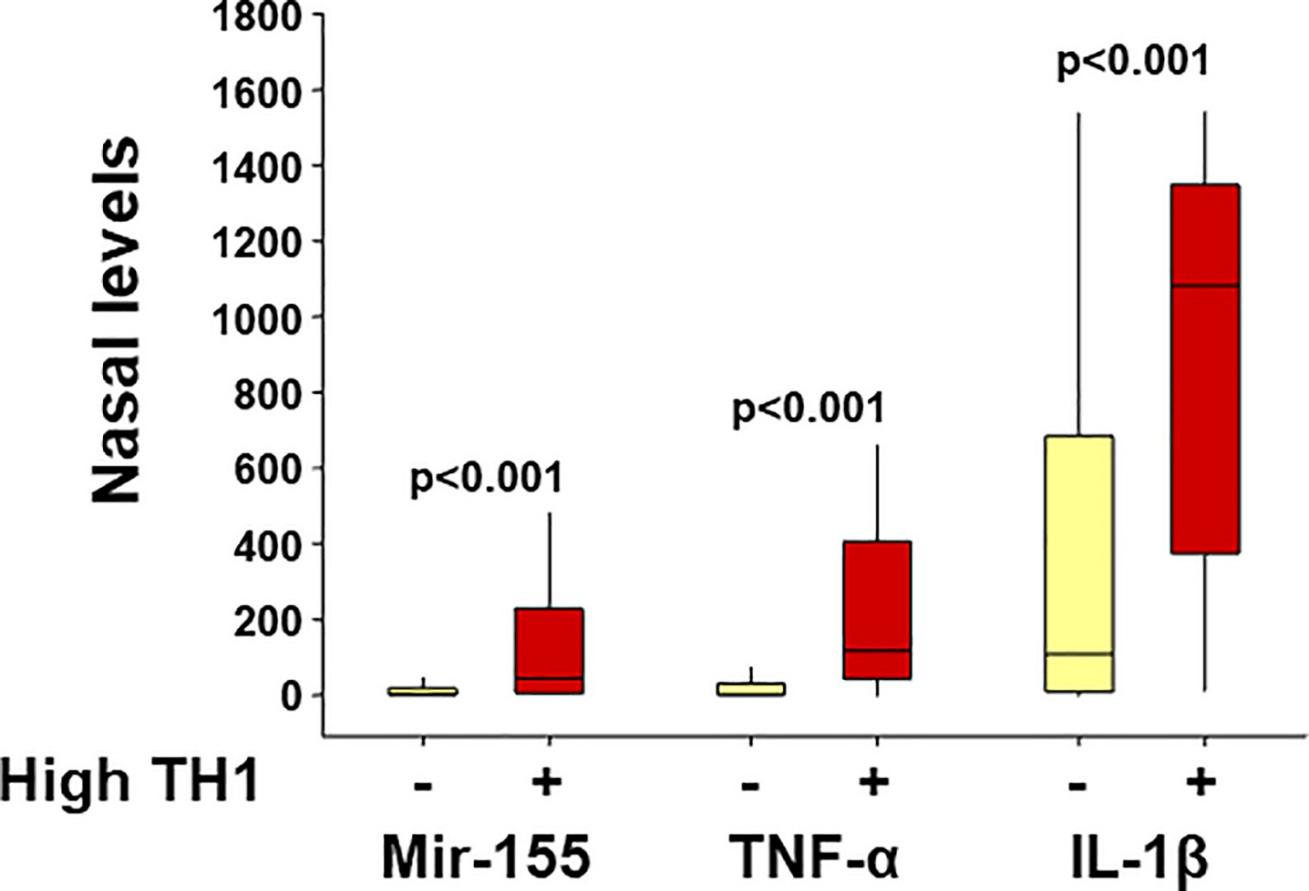
Individuals with a high TH1 polarization profile (IFN-γ:IL-4 ratios >75th%tile; red boxplots; n = 34) have a distinct airway molecular signature characterized by higher nasal miR-155 levels and increased nasal protein levels of pro-inflammatory cytokines (TNF-α, IL-1β) relative to the rest of study subjects (IFN-γ:IL-4 ratios ≤75th percentile; yellow boxplots; n = 106). Nasal levels (y axis) correspond to protein concentration (pg/ml) for TNF-α, IL-1β and to normalized expression for miR-155 (ΔΔCt method normalized to a spike in control cel-miR-39).

To begin to understand the physiological function of miR-155 in the airways during early life, we examined the nasal cytokine responses of the children with viral respiratory infections (n = 140) according to individual miR-155 levels. As shown in Fig 1, we found that those with the highest miR-155 airway levels (>75^th^ percentile) had significantly higher TH1 antiviral cytokine responses (IFN-γ) and increased production of pro-inflammatory (TNF-α, IL-1β) and TH2 cytokines (IL-4, IL-13). Importantly, we also identified that high miR-155 airway levels were strongly associated with increased TH1 cytokine polarization (IFN-γ/IL-4 ratios, Fig 2 or IFN-γ /IL-13 ratios S1 Fig). The subset of subjects with the highest virus-induced TH1 cytokine polarization (IFN-γ/IL-4 ratios >75^th^ percentile; n = 34) also had significantly higher pro-inflammatory responses (TNF-α, IL-1β levels) and increased normalized miR-155 levels (Fig 3). We did not identify a significant difference in IL-13 responses subjects with the highest virus-induced TH1 cytokine polarization (p = 0.08). These data indicate that there is a subset of young children with viral respiratory infections characterized by a distinct airway molecular signature that includes high miR-155 levels and high TH1/pro-inflammatory responses.

### Nasal airway miR-155 levels and clinical severity during viral respiratory infections in young children

We also examined the association between nasal airway miR-155 responses and the severity of viral respiratory infections using respiratory distress scores that included wheezing, retractions, supplemental O2 needs, respiratory rate, and heart rate at the initial presentation (S1 Table). Given that miR-155 and pro-inflammatory nasal airway responses (TNF-α, IL-1β) were significantly different in children with high TH1 polarization (IFN-γ:IL-4 ratios >75^th^ percentile, Fig 3), we subdivided our study subjects according to high TH1 vs. non-high TH1 status. Notably, we found that higher nasal miR-155 levels and higher IFN-γ antiviral responses were significantly associated with lower severity scores independently of age and RSV status in the subset of young children with high TH1 polarization (Table 3). However, this association was not present in the rest of the children with viral respiratory infections (IFN-γ/IL-4 ratios ≤75^th^ percentile; n = 106, Table 3). In individuals with high TH1 polarization we also found that higher severity scores were linked to previously identified risk factors for severe viral respiratory infection in young children [24–27] including preterm status (<37 weeks gestation), black race and RSV status adjusted by younger age (Table 3).

**Table 3:** Airway miR-155, TH1 responses and clinical severity during viral respiratory infections. RV = human rhinovirus, RSV = human respiratory syncytial virus. *High TH1 group defined as IFN-γ:IL-4 ratios >75th%tile, **Regression model adjusted by age and RSV status.

High TH1 group (n = 34)*					Non-high TH1 group (n = 106)				
**Univariate**			Adj. Model**		Univariate			Adj. Model**	
Respiratory severity score	Effect estimate (β)	p-value	Effect estimate (β)	p-value	Respiratory severity score	Effect estimate (β)	p-value	Effect estimate (β)	p-value
**MiR-155**	-0.005	**0.04**	-0.005	**0.04**	**MiR-155**	0.002	0.47	0.002	0.46
**IFN-γ**	-0.0004	**0.02**	-0.0004	**0.02**	**IFN-γ**	-0.0004	0.24	-0.006	0.11
**IL-1β**	0	0.96	0	0.98	**IL-1β**	-0.0003	0.52	-0.0003	0.43
**TNF-α**	-0.0007	0.54	-0.001	0.21	**TNF-α**	-0.0001	0.88	-0.0001	0.82
**Age**	-1.31	0.15	-1.64	**0.04**	**Age**	-0.58	0.14	-0.53	0.18
**Sex**	-1.13	0.32	-0.73	0.49	**Sex**	0.73	0.28	0.71	0.29
**Black race**	3.31	**0.002**	3.0	**0.004**	**Black race**	-1.005	0.123	-0.96	0.14
**Preterm**	3.8	**0.001**	3.07	**0.006**	**Preterm**	0.961	0.138	1.27	0.05
**RV**	0.90	0.44	1.28	0.28	**RV**	-1.2	0.07	-1.2	0.06
**RSV**	2.60	0.09	3.49	**0.02**	**RSV**	0.92	0.28	0.78	0.36

## Discussion

This study provides novel human-based molecular insights about the regulation of antiviral airway responses during early life. We identified that miR-155 is increased in the nasal aspirates of young children (≤2 years) during viral infections independent of the causative respiratory virus. We also examined for the first time the *in vivo* correlation of nasal miR-155 responses, individual airway cytokine profiles, and clinical severity during naturally occurring viral respiratory infections in young children. The importance of these data is that miR-155 is an evolutionarily-conserved molecule with essential but still unclear roles promoting both TH1 antiviral immunity [3–12], as well as TH2 allergic inflammation [16–19]. Thus, there is increasing interest in defining the precise role(s) of miR-155 in human airway immunity and in the pathobiology of airway diseases such as COPD [23], asthma [28], and viral respiratory infections in young children [14,15].

Over the past decade many studies have identified that miR-155 is a master regulator of the immune system [1,2]. Indeed, there is strong evidence that miR-155 controls CD8+ and CD4+ T cell activation, proliferation, and cytokine production *in vitro* and *in vivo* during viral infection (8–10). MiR-155 also plays a crucial role in the generation of protective adaptive immune responses in B-cells, including antigen presentation and antibody production [3]. Several studies have reported that miR-155 is essential for the generation of TH2-mediated inflammation as well [16–19]. Dendritic cells [16] and type 2 innate lymphoid cells [19] derived from miR-155(-/-) KO mice have limited TH2 priming capacity.Additionally,CD4+ TH2 cells require intrinsic miR-155 expression for type-2 immune polarization [18]. This experimental evidence has led to the notion that miR-155 may be a potential target to alleviate TH2-driven conditions, including allergic asthma [17,28]. Nonetheless, most of this evidence is derived from animal models and the physiological function of miR-155 in the human airways during health and disease is still poorly understood. Filling this void is critically necessary before pursuing miR-155-based clinical applications. Our current study begins to address this gap with new data about the human airway miR-155 responses during viral respiratory infections in early life. Specifically, we identified that high miR-155 levels are associated with increased production of TH1 (IFN-γ) and TH2 cytokines (IL4, IL-13) in the nasal airways of young children with viral respiratory infections. Although miR-155 was linked to smaller changes in IL-4 and IL-13 (Fig 1), it is possible that subtle changes may be biologically relevant for allergic inflammation. Thus, these results are in line with the reported dual role of miR-155 promoting TH1 (antiviral) [3–12] and TH2 (allergic) responses [16–19]. However, in our study we also identified that high miR-155 levels were primarily associated with enhanced TH1 cytokine polarization (greater IFN-γ/IL-4 ratios, Fig 2), indicating that, although miR-155 likely has several effects in the airways, the overall physiological role of miR-155 in human infants and young children appears to be the upregulation of host antiviral immunity during viral respiratory infections.

MiR-155 is also a robust marker of pro-inflammatory responses in different cell systems [20–23]. There is compelling evidence demonstrating that miR-155 is highly expressed in macrophages, dendritic cells, as well as T and B lymphocytes [1–3] and is selectively exported by these cells in exosomes to allow cell-to-cell communication and amplification of pro-inflammatory responses in the lungs [29]. Thus we believe that the association between high miR-155 levels and TH1 cytokine polarization in our study is likely the result of multiple processes during viral infections: 1) the secretion of exosomes containing miR-155 triggered by respiratory viruses [14,29]; 2) the effects of miR-155 promoting pro-inflammatory responses and a TH1 bias [3–12]; and 3) the positive feedback generated by TH1 and other pro-inflammatory cytokines that induce miR-155 production [7,30]. Together, these processes may lead to an autocrine/paracrine loop in the airways to promote TH1 polarization in individuals with high miR-155 levels during viral respiratory infections. Nonetheless, there are alternative explanations for the link between miR-155 and TH1 responses. For instance, given that viral loads were not obtained in this study, it is also possible that the production of miR-155 could have been driven by higher viral replication. Thus the production of miR-155 and IFN-y (as well as other cytokines) might be similarly upregulated by another factor (such as viral load or timing of infection). In addition, it is important to clarify that our findings only pertain to the nasal airway mucosa since respiratory viruses may be acting differently in nasal or bronchial cells inducing miRNA-155.

Our results also suggest that in the setting of a viral respiratory infection, the interplay between miR-155 and TH1 responses may promote additional pro-inflammatory responses in the airways. Specifically, we found that: 1) young children with high miR-155 nasal levels during viral infection had increased production of pro-inflammatory cytokines (TNF-α, IL-1β); and 2) the individuals with the highest levels of TH1 cytokine polarization had higher TNF-α and IL-1β levels. These data indicate that there is a subset of young children with viral respiratory infections characterized by high miR-155 levels and high TH1/pro-inflammatory responses. Notwithstanding the compelling evidence linking miR-155 with pro-inflammatory responses [20–23], it is relevant to note that the mechanisms by which miR-155 controls inflammation are complex and appear to be cell specific due to context-dependent miR-155 targeting and regulation of gene expression [5,6]. Certainly, there is compelling evidence demonstrating that in bone marrow-derived immune cells (e.g. macrophages), miR-155 potentiates NF-κB activity and induces the production of pro-inflammatory cytokines (e.g. TNF-α) via suppression of SHIP1 and SOCS1 (miR-155 targets) [21]. Conversely, studies in endothelial cells have identified that miR-155 can inhibit NF-kB-driven inflammation by targeting RELA (p65/NF-kB) directly [31]. Indeed, RELA contains highly conserved miR-155-binding sites in its 3'-UTR, and experimental evidence (gene reporter assays) has confirmed that miR-155 can bind to these target sites to suppress NF-kB activation in endothelial cells [31]. Other studies have reported additional cytoprotective effects of miR-155 on these cells [32,33]. Thus, we believe that additional studies are needed to examine the actions of miR-155 in a cell specific and context-dependent manner because miR-155 targets and companion functional outcomes may vary across different human airway cell types and in the presence of different immune and inflammatory mediators.

In concert with the above observations, it is noteworthy that in our study the association between clinical severity and miR-155 responses during viral infections was context-dependent and varied according to the individual airway TH1 responses. Specifically, we found that higher miR-155 levels were associated with lower respiratory disease severity in the subset of children with the highest TH1 responses but not in the rest of children (Table 2). In children with high TH1 polarization, we also observed that less clinical severity was linked to a higher production of IFN-γ but not of other cytokines associated high miR-155 levels (TNF-α, IL-1β, Fig 3 and Table 2). Collectively, these findings suggest that the evolutionarily-conserved function of miR-155 in the airways of human infants is primarily to provide host protection by enhancing antiviral immunity during early life (e.g. TH1 responses). Nonetheless, it is possible that the airway production of miR-155 in different contexts (e.g. smoke inhalation) may amplify inflammatory responses and lead to greater respiratory disease severity [23], particularly in individuals prone to develop severe airway inflammatory responses (e.g. asthma or COPD) [23,28].

In conclusion, the present study examined the airway production of miR-155 in young children during *in vivo* viral respiratory infections. The results provide new evidence that miR-155 is strongly linked to high IFN-γ production and enhanced airway TH1 cytokine polarization (IFN-γ/IL-4 ratios). High airway miR-155 levels were linked to decreased respiratory disease severity in individuals with high TH1 antiviral responses. These observations are consistent with the collection of evidence implicating crucial roles for miR-155 in regulating antiviral airway responses in several animal models and cell systems [3–23]. Our current results demonstrate that miR-155 is also relevant to the immunobiology of the human airways during early life, and they highlight the need to conduct additional studies to better define the physiological role(s) of the secretory responses of miR-155 in the respiratory tract of human infants and young children.

## Supporting information

S1 Fig Nasal miR-155 levels stratified as high (>75th%ile), medium (25-75th%ile), or low (<25th%ile) demonstrate that young children with high miR-155 levels during viral respiratory infections have higher airway TH1 cytokine polarization (IFN-γ:IL-13ratios). (DOCX)

S1 Table Respiratory distress score. (DOCX)
