# Supplementary material for: Airway mir-155 responses are associated with TH1 cytokine polarization in young children with viral respiratory infections
Source: PLoS One. 2020 May 22;15(5):e0233352. doi: 10.1371/journal.pone.0233352 (PMC7244143; doi:10.1371/journal.pone.0233352)
Supplement: S1 Fig — (DOCX) [file pone.0233352.s001.docx]

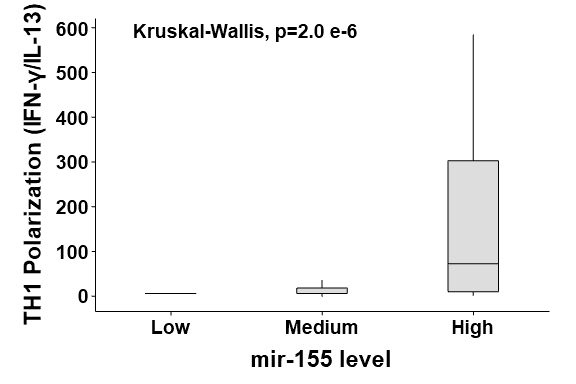


**Figure S1.** Nasal miR-155 levels stratified as high (>75th%ile), medium (25-75th%ile), or low (<25th%ile) demonstrate that young children with high miR-155 levels during viral respiratory infections have higher airway TH1 cytokine polarization (IFN-γ:IL-13ratios).
