# Supplementary material for: Airway mir-155 responses are associated with TH1 cytokine polarization in young children with viral respiratory infections
Source: PLoS One. 2020 May 22;15(5):e0233352. doi: 10.1371/journal.pone.0233352 (PMC7244143; doi:10.1371/journal.pone.0233352)
Supplement: S1 Table — (DOCX) [file pone.0233352.s002.docx]

**Table S1. Respiratory distress score**

| Score | **Sub-costal retractions** | **Wheezing** | **Respiratory rate** | **Heart rate** | **Supplemental O2** |
| --- | --- | --- | --- | --- | --- |
| 0 | **None** | **None** | **<30 bpm** | **<120 bpm** | **None** |
| 1 |  | **Mild** | **30-45 bpm** | **120-160 bpm** |  |
| 2 | **(+)** |  | **45-60 bpm** | **> 160 bpm** | **(+)** |
| 3 |  | **Prominent** | **>60 bpm** |  |  |
